# Impact of Early Rehabilitation on Functional Recovery After Kidney Transplantation: A Retrospective Cohort Study

**DOI:** 10.7759/cureus.91046

**Published:** 2025-08-26

**Authors:** Masashi Ichikawa, Yuki Uchiyama, Norihiko Kodama, Shinya Yamauchi, Yusuke Yamada, Michio Nojima, Shingo Yamamoto, Kazuhisa Domen

**Affiliations:** 1 Department of Rehabilitation Medicine, School of Medicine, Hyogo Medical University, Nishinomiya, JPN; 2 Department of Physical Therapy, School of Rehabilitation, Hyogo Medical University, Kobe, JPN; 3 Department of Rehabilitation, Hyogo Medical University Hospital, Nishinomiya, JPN; 4 Department of Urology and Kidney Transplant Center, School of Medicine, Hyogo Medical University, Nishinomiya, JPN

**Keywords:** early rehabilitation, frailty, kidney transplantation, renal rehabilitation, unsupervised home training

## Abstract

Background: The trajectory of recovery following early rehabilitation after kidney transplantation remains poorly understood, particularly in relation to preoperative frailty. This study aimed to examine how physical function and health-related quality of life (HRQOL) recover over time after kidney transplantation with early rehabilitation. In particular, we assessed whether recovery patterns differed between robust and non-robust (pre-frail/frail) patients.

Methods: We retrospectively analyzed 15 living-donor kidney transplant recipients (median age: 45 years) who underwent transplantation between April 1, 2023, and June 18, 2025. All patients received early rehabilitation and functional assessments in the preoperative period and at one, two, and four months after transplantation. Frailty was assessed using the revised Japanese version of the Cardiovascular Health Study criteria, classifying patients into a robust group (n = 7) and a non-robust (pre-frailty and frailty) group (n = 8). The rehabilitation program included early mobilization, aerobic exercise, resistance exercise combined with virtual reality-based core training, and unsupervised home training. Measures included body composition, physical function (e.g., 6-minute walk distance (6MWD), gait speed, and muscle strength), HRQOL, and physical activity. Baseline characteristics were compared using t-tests or Fisher’s exact test, and longitudinal as well as between-group differences were assessed using repeated-measures ANOVA with Bonferroni correction, with p < 0.05 considered statistically significant.

Results: In the overall cohort, there was a significant decrease in body weight postoperatively (p < 0.05), with significant improvements in 6MWD, gait speed, the Timed Up & Go test, knee extensor strength, the EuroQol 5-Dimension 5-Level index, the EuroQol visual analogue scale, and sedentary time observed by four months after transplantation (all p < 0.05). Both groups showed functional improvements. The robust group demonstrated earlier improvements and tended to show greater longitudinal gains in 6MWD over time (p = 0.054). Early rehabilitation had no adverse effects on graft function.

Conclusions: Early rehabilitation after kidney transplantation improved physical function and HRQOL over four months, with benefits observed regardless of frailty status. Continued home training further supported recovery; however, the retrospective single-center design with a small sample size, short follow-up, variable adherence, and lack of a control group represent limitations.

## Introduction

The proportion of elderly individuals is increasing rapidly in most advanced countries. Consequently, the number of patients with chronic kidney disease (CKD) is also rising. In Japan, the estimated number of individuals with CKD was approximately 15 million in 2015 [1]. A considerable proportion of these patients progress to end-stage kidney disease, necessitating kidney transplantation. A total of 1,782 kidney transplantations were performed in Japan in the 2022 fiscal year [2].

Frailty is also a common health-related concern in aging societies. It is well recognized that aging is one of the most important factors contributing to frailty. In addition to aging, impaired renal function, which can lead to malnutrition, uremia, chronic inflammation, osteoporosis, cardiovascular disorders, and insulin resistance, has been identified as an independent risk factor for frailty [3,4]. Furthermore, reduced physical function, including frailty, is associated with increased mortality [5]. In light of these factors, rehabilitation is often prescribed for patients who have undergone kidney transplantation in order to prevent the progression of frailty.

The existing literature indicates that rehabilitation improves exercise tolerance and quality of life in patients following kidney transplantation [6]. However, such improvements are typically observed in patients who are more than a few months out from transplantation, rather than in the immediate postoperative period. One study found that the prevalence of frailty was 19.8% among patients awaiting kidney transplantation, increasing further at one month after surgery and then decreasing by three months after transplantation [7]. Importantly, Yamamoto et al. reported that patients who started rehabilitation soon after surgery showed improvements in exercise tolerance and knee extension strength at two months after transplantation [8]. However, the number of studies investigating the time course of recovery following rehabilitation remains limited. Moreover, few studies have examined the impact of pre-existing frailty before surgery. In this study, we examined the time course to recovery of physical functions, such as exercise tolerance, following rehabilitative intervention, taking into account pre-existing frailty before surgery.

## Materials and methods

Study design and setting

This retrospective observational study included kidney transplant recipients who underwent living-donor kidney transplantation at Hyogo Medical University Hospital between April 1, 2023, and June 18, 2025, and participated in early postoperative rehabilitation. Patients were included in the study if they completed functional assessments in the preoperative period and at one, two, and four months after transplantation. In accordance with previous studies on post-transplantation outcomes, patients were excluded from the final analysis if they had a severe physical condition unrelated to CKD, such as lumbar spinal canal stenosis or severe joint pain in the lower extremities, that could affect assessments of physical function, including the 6-minute walking distance (6MWD) and other mobility-related tests. The study protocol was approved by the Hyogo Medical University Research Ethics Committee (approval number: 5057 (202507-054)). Informed consent was obtained using the opt-out route via the hospital’s website.

Early rehabilitation program after kidney transplantation

**Figure 1:**
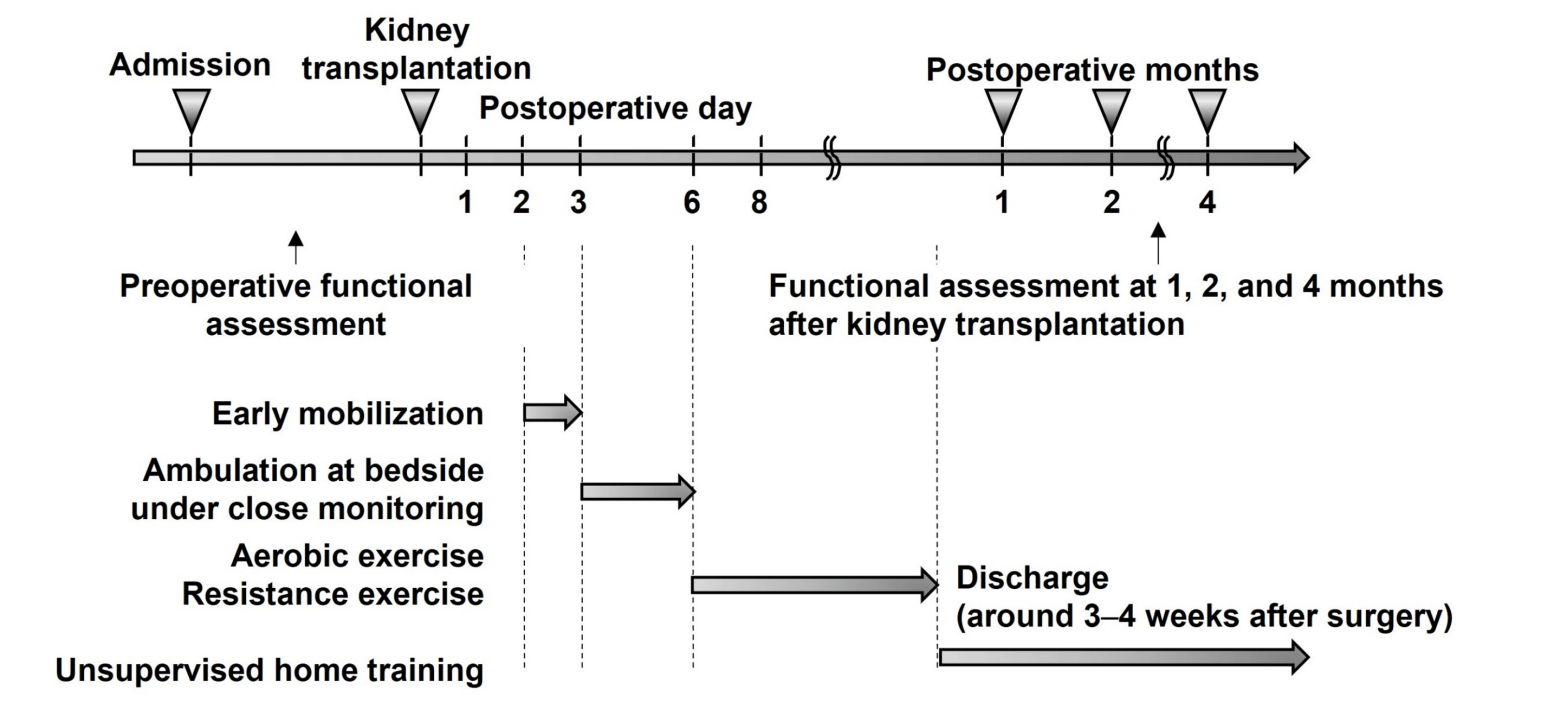
Early rehabilitation program after kidney transplantation A structured rehabilitation program was started in the early postoperative phase. Functional assessments were performed preoperatively and at one, two, and four months after transplantation. Mobilization was started on postoperative day 2, followed by bedside ambulation on postoperative day 3. If the postoperative course was stable, aerobic and resistance exercises were initiated between postoperative days 6 and 8 and continued until discharge (around three to four weeks after surgery). After discharge, unsupervised home training was introduced and maintained throughout the follow-up period.

Figure 1 shows the structured rehabilitation program implemented in the early phase after kidney transplantation. Functional assessments were systematically scheduled for the preoperative period and at one, two, and four months after transplantation. Early mobilization, including sitting and standing, was initiated from postoperative day 2. By postoperative day 3, standing and gait training was initiated at the bedside under strict medical supervision. If patients remained stable in terms of hemodynamics and respiratory status, individualized exercise therapy was commenced between postoperative days 6 and 8 and continued throughout hospitalization.

Our rehabilitation program consisted of three major components, namely aerobic exercise, resistance exercise combined with virtual reality-based core training, and unsupervised home training after discharge. The details of these components are as follows. Aerobic exercise was performed four or five times per week at a moderate intensity, maintained at 12-13 on the Borg’s Rating of Perceived Exertion scale and not exceeding 15. Each session lasted 30-40 minutes, with a minimum duration of 20 minutes, and was performed in one to two sets depending on individual exercise tolerance. Physical activities included walking or using an ergometer device, which allowed adjustment of workload (watts).

Resistance exercise, including bodyweight exercises focused on the core and gluteal muscles, was also conducted four or five times per week. Each set consisted of 10-15 repetitions, and the number of sets was adjusted depending on individual exercise tolerance. In addition, virtual reality-based core training was performed using a mediVR KAGURA® device (mediVR Inc., Osaka, Japan) for approximately 20 minutes per session, three times per week, while monitoring for postoperative pain and discomfort. The program consisted of seated reaching movements toward virtual targets displayed in the environment to both sides while wearing a head-mounted display and has been reported to enhance core stability and gait performance [9].

Unsupervised home training was initiated after discharge. Patients were instructed to walk daily, with a target of at least 6,000 steps per day, and preferably more than 10,000 steps, monitored using a pedometer (FB-740, Tanita Co., Ltd., Tokyo, Japan). They were also encouraged to continue home-based resistance exercises, such as calf raises and squats, depending on individual exercise tolerance. The exercise program was implemented safely and appropriately in accordance with the discontinuation criteria suggested by Yamamoto et al. [8].

Measurements

During the study period, patients were evaluated during the preoperative period and at one, two, and four months after transplantation. Demographic and clinical characteristics, including age, sex, body weight, dialysis duration, the underlying renal diagnosis, comorbidities, and laboratory data, were collected from the electronic medical records. The comorbidity burden was quantified using the Charlson Comorbidity Index (CCI), which is a validated measure of disease severity [10].

Laboratory measurements included creatinine (Cre, mg/dL), hemoglobin (Hb, g/dL), albumin (Alb, g/dL), and estimated glomerular filtration rate (eGFR, mL/min/1.73 m²), which was calculated using Cre. Body composition was assessed using a multifrequency bioelectrical impedance analysis (BIA) system (InBody S10, InBody Japan Inc., Tokyo, Japan) to measure the skeletal muscle mass index (SMI) and body fat percentage. SMI was calculated as follows: SMI = appendicular skeletal muscle mass (kg)/height (m)².

Physical function was evaluated using the 6MWD, gait speed (usual), the Timed Up & Go test (TUG), grip strength, and knee extension strength. The 6MWD, widely used as a primary outcome measure of exercise tolerance in patients with CKD or chronic heart failure, was assessed in accordance with the American Thoracic Society (ATS) guidelines [11]. Patients were instructed to walk back and forth along a 20-m corridor for six minutes at their own pace, encouraged to cover as much distance as possible, but allowed to rest and resume walking or to stop if they experienced dyspnea or leg pain. Gait speed was measured over a 16-m walkway, which included a 10-m timed section and 3-m acceleration and deceleration zones on each side. The TUG, a measure of dynamic balance, was performed in accordance with the method proposed by Podsiadlo and Richardson [12]. In this test, the patient was timed while rising from an armchair, walking 3 m, turning around, walking back, and sitting down again. Grip strength was assessed using a standardized dynamometer with an adjustable handle (TKK 5101, Takei Scientific Instruments Co. Ltd., Niigata, Japan), with two trials per hand; the average of the highest values from both hands was used for analysis. Knee extension strength was measured using a hand-held dynamometer (µTas MT-1, ANIMA Co., Tokyo, Japan) in a seated position with the knee flexed at approximately 90°. The device was fixed to the distal lower leg, and the patient was instructed to perform maximal isometric contractions for 10 s. The knee extension strength-to-body weight ratio was calculated as follows: knee extension strength (kgf)/body weight (kg). Each leg was tested twice, and the maximum value was used for analysis.

Health-related quality of life (HRQOL) was assessed using the EuroQol 5-Dimension 5-Level (EQ-5D-5L) questionnaire and the EuroQol visual analogue scale (EQ-VAS). The EQ-5D-5L consists of five domains (mobility, self-care, usual activities, pain/discomfort, and anxiety/depression), each of which is rated on a five-point scale [13]. Each item is answered on a five-point scale, and index values are calculated using an algorithm. The EQ-VAS records the patient’s self-rated health on a scale from 0 (worst imaginable health) to 100 (best imaginable health). Validated Japanese versions of both questionnaires were used [14].

Physical activity was measured using the International Physical Activity Questionnaire-Short Form (IPAQ, Japanese version) [15,16]. Activity levels were quantified in metabolic equivalents (METs). In accordance with the IPAQ scoring guidelines, physical activity for each category was calculated as follows: MET value × minutes per day × days per week. Total physical activity was defined as the sum of these values. Sedentary behavior was also assessed using a standard item in the IPAQ to quantify daily sedentary time.

Diagnosis of frailty based on the revised J-CHS criteria

Frailty status was assessed using the revised Japanese version of the Cardiovascular Health Study (J-CHS) criteria [17], based on the classification system proposed by Fried et al. [18]. Patients were initially categorized into robust, pre-frailty, and frailty groups. Frailty was defined as meeting three or more of the following five criteria: (1) unintentional weight loss of ≥2 kg within six months, (2) self-reported exhaustion, (3) low physical activity, (4) weakness, defined as grip strength <28 kg for men and <18 kg for women, and (5) slowness, defined as usual walking speed <1.0 m/s. Individuals meeting one or two criteria were categorized as having pre-frailty, while those meeting none of these criteria were considered robust. For analysis, we combined the patients with pre-frailty and frailty into a non-robust group, and those with a score of 0 were classified as the robust group. This approach has been effectively applied in studies of patients with CKD [19].

Statistical analysis

Baseline characteristics are reported as the frequency (percentage) if categorical and as the median with the interquartile range (IQR) if continuous. Data were compared between the robust and non-robust groups using unpaired t-tests for continuous variables and Fisher’s exact test for categorical variables. Longitudinal changes in continuous variables, including body weight, SMI, body fat percentage, Cre, eGFR, Hb, Alb, 6MWD, gait speed, TUG, grip strength, knee extension strength, EQ-5D-5L index values, EQ-VAS score, physical activity, and sedentary time, were evaluated using one-way repeated measures analysis of variance (ANOVA). The analysis was performed across four time points (preoperative period and one, two, and four months after transplantation) with time treated as a within-subject factor. Post hoc comparisons (preoperative period vs. one, two, and four months) were adjusted using Bonferroni correction. For variables showing significant longitudinal changes, two-way repeated measures ANOVA was used to assess between-group differences (robust vs. non-robust) as a between-subject factor and to examine their interaction effect with time. There were no missing data in this study. Although the distribution of some variables was non-normal, ANOVA was applied considering its robustness to deviations from normality. All analyses were conducted using EZR software (Saitama Medical Center, Jichi Medical University, Saitama, Japan), with p-values < 0.05 considered statistically significant.

## Results

**Figure 2:**
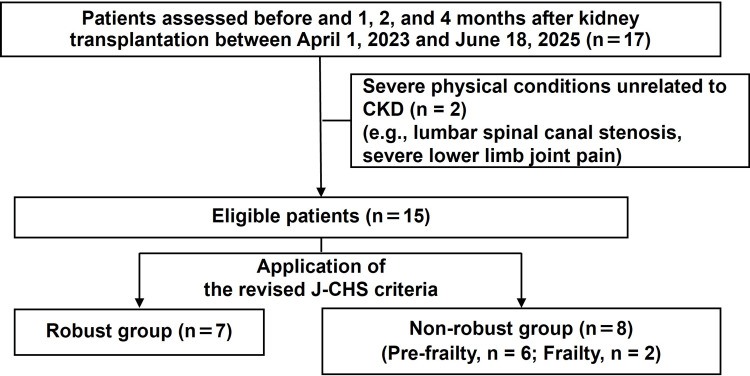
Patient flow diagram Seventeen patients who completed all scheduled assessments were enrolled. Two patients were subsequently excluded because of severe physical impairments unrelated to CKD that could have affected the results of assessments of physical function. The final analysis included 15 patients (median age, 45 years), who were classified into a robust group (n = 7) and a non-robust group (n = 8; 6 pre-frail, 2 frail) based on the revised J-CHS criteria. CKD, chronic kidney disease; J-CHS, Japanese version of the Cardiovascular Health Study.

Figure 2 shows a flowchart of patients in the study. Patients were evaluated in the preoperative period and at one, two, and four months after transplantation. A total of 17 patients completed all scheduled assessments and were enrolled in the study. Of these patients, two were subsequently excluded because of severe physical impairments unrelated to CKD, such as lumbar spinal canal stenosis or severe joint pain in the lower extremities, which could have affected assessments of physical function. The final sample consisted of 15 patients with a median age of 45 years. Based on the revised J-CHS criteria, there were seven patients (46.7%) in the robust group and eight (53.3%) in the non-robust group. The non-robust group included six patients (40.0%) who were pre-frail and two (13.3%) who were frail. In this study, early rehabilitation was provided throughout hospitalization, with a median length of stay from kidney transplantation to discharge of 28.0 days (IQR, 22.0-29.0). In addition, when stratified by group, the median length was 21.0 days (IQR, 21.0-23.5) in the robust group and 29.0 days (IQR, 28.8-32.5) in the non-robust group.

Table 1 summarizes the baseline characteristics of the two groups. Compared with the robust group, the non-robust group included significantly fewer male patients and patients with significantly lower body weight and SMI. There were no significant differences between the groups in dialysis duration, the underlying renal diagnosis, or laboratory data. However, the CCI was significantly higher in the non-robust group. Grip strength was also significantly lower in the non-robust group. There were no significant between-group differences in other measures of physical function, including EQ-5D-5L index values, EQ-VAS, physical activity, and sedentary time. Nevertheless, the non-robust group generally tended to show lower values across these measures.

**Table 1:** Patient demographics and clinical characteristics The data are presented as the median (interquartile range) or frequency (percentage). The p-values represent comparisons between the robust and non-robust groups. Statistically significant findings (p < 0.05) are marked with an asterisk. 6MWD, 6-minute walking distance; Alb, albumin; ADPKD, autosomal dominant polycystic kidney disease; CCI, Charlson comorbidity index; Cre, creatinine; eGFR, estimated glomerular filtration rate; EQ-5D-5L, EuroQol 5-Dimension 5-Level; EQ-VAS, EuroQol visual analogue scale; Hb, hemoglobin; IgA, immunoglobulin A; IPAQ, International Physical Activity Questionnaire-Short Form; METs, metabolic equivalents; SMI, skeletal muscle mass index; TUG, Timed Up & Go test.

Variable	All patients (n = 15)	Robust group (n = 7)	Non-robust group (n = 8)	t-value	p-value
Age, years	45.0 (41.5, 50.5)	42.0 (41.0, 45.0)	50.5 (44.3, 62.3)	2.074	0.059
Male sex, %	7.0 (46.7)	6.0 (85.7)	1.0 (12.5)		<0.05*
Body weight, kg	66.6 (54.3, 71.3)	70.8 (63.6, 84.0)	54.9 (51.7, 66.7)	-2.388	<0.05*
SMI, kg/m^2^	7.6 (6.1, 8.6)	8.8 (8.3, 8.9)	6.4 (5.6, 7.4)	-3.319	<0.01*
Body fat percentage, %	23.3 (21.0, 28.4)	22.7 (18.8, 23.2)	28.4 (23.4, 31.9)	1.956	0.072
Duration of dialysis, months	10.0 (0.25, 20.5)	10.0 (0.0, 24.8)	11.5 (6.1, 17.3)	-0.613	0.551
Underlying renal diagnosis, %					
Nephrosclerosis	2.0 (13.3)	0.0 (0.0)	2.0 (25.0)		0.155
IgA nephropathy	5.0 (33.3)	2.0 (28.6)	3.0 (37.5)		0.714
ADPKD	4.0 (26.7)	2.0 (28.6)	2.0 (25.0)		0.876
CCI	3.0 (3.0, 4.5)	3.0 (2.5, 3.0)	4.5 (3.0, 6.0)	2.222	<0.05*
Laboratory data					
Cre, mg/dL	11.6 (11.1, 13.2)	13.4 (11.9, 14.6)	11.5 (10.6, 11.7)	-1.346	0.201
eGFR, mL/min/1.73 m^2^	4.0 (3.0, 4.0)	4.0 (3.5, 4.0)	3.5 (3.0, 4.0)	-0.286	0.779
Hb, g/dL	10.6 (10.2, 12.0)	11.1 (10.2, 12.0)	10.6 (10.1, 12.0)	-0.134	0.895
Alb, g/dL	3.8 (3.6, 4.2)	3.8 (3.6, 4.2)	3.9 (3.6, 4.1)	-0.424	0.678
Physical performance					
6MWD, m	513.0 (395.5, 536.5)	520.0 (420.0, 581.0)	465.5 (395.0, 523.3)	-1.209	0.248
Gait speed (usual), m/s	1.24 (1.20, 1.41)	1.40 (1.23, 1.43)	1.22 (1.03, 1.27)	-1.964	0.071
TUG, seconds	7.13 (6.50, 8.11)	6.44 (6.38, 6.95)	7.59 (7.06, 9.63)	1.848	0.087
Grip strength, kg	25.7 (22.7, 35.0)	35.1 (34.8, 39.3)	22.7 (19.6, 24.0)	-4.348	<0.01*
Knee extension strength, kgf/kg	0.49 (0.41, 0.60)	0.52 (0.44, 0.64)	0.49 (0.38, 0.53)	-1.325	0.208
EQ-5D-5L index value	0.867 (0.797, 0.947)	0.867 (0.845, 1.000)	0.826 (0.761, 0.895)	-1.174	0.262
EQ-VAS	70.0 (62.5, 80.0)	80.0 (67.5, 85.0)	67.5 (63.8, 70.0)	-1.403	0.184
IPAQ, METs·minutes/week	528.0 (393.5, 1683.0)	990.0 (429.0, 4905.0)	462.0 (367.5, 945.8)	-1.828	0.091
Sedentary time, minutes	540.0 (360.0, 840.0)	360.0 (210.0, 630.0)	780.0 (465.0, 900.0)	1.979	0.069

Table 2 compares the longitudinal change (a within-subject factor) in clinical and functional measures across all participants. One-way repeated measures ANOVA revealed significant longitudinal changes (with p-values ranging from < 0.01 to < 0.05) in body weight, SMI, Hb, Alb, 6MWD, gait speed, TUG, knee extension strength, EQ-5D-5L index values, EQ-VAS, and sedentary time. In the post hoc comparisons, significant improvements in Alb, 6MWD, TUG, and knee extension strength were observed between the preoperative period and two months after transplantation. Significant improvements were also noted in 6MWD, gait speed, TUG, knee extension strength, EQ-5D-5L index values, EQ-VAS, and sedentary time between the preoperative period and four months after transplantation. Body weight showed a consistent significant decrease throughout the postoperative period. SMI showed a significant reduction only at one month post-transplantation and remained stable thereafter. Although Hb showed significant longitudinal changes, there were no significant differences in comparisons between the preoperative period and each postoperative time point. However, Hb showed a marked decline at one month after transplantation. In terms of renal function, both Cre and eGFR showed significant postoperative improvement and remained stable throughout the observation period.

**Table 2:** Within-subject changes in functional measures over four months after kidney transplantation Data are presented as the median with the interquartile range. The p-values represent longitudinal changes (within-subject factor) in functional measures across all participants, including post hoc comparisons. Statistically significant findings (p < 0.05) are marked with asterisks. 6MWD, 6-minute walking distance; Alb, albumin; Cre, creatinine; eGFR, estimated glomerular filtration rate; EQ-5D-5L, EuroQol 5-Dimension 5-Level; EQ-VAS, EuroQol visual analogue scale; Hb, hemoglobin; IPAQ, International Physical Activity Questionnaire-Short Form; IQR, interquartile range; METs, metabolic equivalents; POM, postoperative month; Pre, preoperative period; SMI, skeletal muscle mass index; TUG, Timed Up & Go test.

		All patients (n = 15)				Longitudinal changes			Post hoc comparisons	
Variable		Pre	POM1	POM2	POM4	F-value	df	p-value	Pre vs POMs	p-value
Body weight, kg	Median	66.6	59.6	60.3	59.0	14.89	3, 42	<0.01*	Pre vs POM1	<0.01*
	IQR	54.3, 71.3	48.9, 68.3	49.5, 68.0	50.3, 70.0				Pre vs POM2	<0.01*
									Pre vs POM4	<0.05*
SMI, kg/m^2^	Median	7.6	7.0	7.2	7.5	4.68	3, 42	<0.05*	Pre vs POM1	<0.01*
	IQR	6.1, 8.6	5.7, 7.7	6.0, 8.6	5.7, 8.0				Pre vs POM2	1.000
									Pre vs POM4	0.390
Body fat percentage, %	Median	23.3	22.8	20.4	22.4	1.78	3, 42	0.166	Pre vs POM1	1.000
	IQR	21.0, 28.4	20.2, 27.0	17.8, 25.3	18.9, 24.8				Pre vs POM2	0.740
									Pre vs POM4	1.000
Laboratory data										
Cre, mg/dL	Median	11.6	1.4	1.3	1.3	171.41	3, 42	<0.01*	Pre vs POM1	<0.01*
	IQR	11.1, 13.2	0.9, 1.6	0.9, 1.5	0.9, 1.6				Pre vs POM2	<0.01*
									Pre vs POM4	<0.01*
eGFR, mL/min/1.73 m^2^	Median	4.0	46.0	47.0	45.0	171.00	3, 42	<0.01*	Pre vs POM1	<0.01*
	IQR	3.0, 4.0	35.0, 51.5	36.0, 54.5	37.0, 51.5				Pre vs POM2	<0.01*
									Pre vs POM4	<0.01*
Hb, g/dL	Median	10.6	9.9	10.8	11.1	4.20	3, 42	<0.01*	Pre vs POM1	0.184
	IQR	10.2, 12.0	9.0, 10.5	10.3, 11.5	10.3, 12.1				Pre vs POM2	1.000
									Pre vs POM4	1.000
Alb, g/dL	Median	3.8	4.1	4.3	4.2	5.32	3, 42	<0.01*	Pre vs POM1	1.000
	IQR	3.6, 4.2	3.9, 4.2	4.1, 4.5	4.0, 4.5				Pre vs POM2	<0.05*
									Pre vs POM4	0.132
Physical performance										
6MWD, m	Median	513.0	547.0	586.0	574.0	11.93	3, 42	<0.01*	Pre vs POM1	1.000
	IQR	395.5, 536.5	420.0, 568.5	493.5, 615.5	469.0, 619.5				Pre vs POM2	<0.01*
									Pre vs POM4	<0.01*
Gait speed (usual), m/s	Median	1.24	1.37	1.33	1.44	7.07	3, 42	<0.01*	Pre vs POM1	1.000
	IQR	1.20, 1.41	1.22, 1.48	1.23, 1.60	1.34, 1.62				Pre vs POM2	0.143
									Pre vs POM4	<0.01*
TUG, seconds	Median	7.13	6.3	6.12	5.98	4.38	3, 42	<0.01*	Pre vs POM1	1.000
	IQR	6.50, 8.11	5.93, 8.29	5.85, 6.77	5.61, 7.06				Pre vs POM2	<0.05*
									Pre vs POM4	<0.01*
Grip strength, kg	Median	25.7	27.5	29.0	26.6	0.48	3, 42	0.695	Pre vs POM1	1.000
	IQR	22.7, 35.0	20.7, 35.8	22.0, 36.1	22.6, 35.2				Pre vs POM2	1.000
									Pre vs POM4	1.000
Knee extension strength, kgf/kg	Median	0.49	0.54	0.55	0.57	9.61	3, 42	<0.01*	Pre vs POM1	1.000
	IQR	0.41, 0.60	0.43, 0.61	0.51, 0.67	0.47, 0.70				Pre vs POM2	<0.01*
									Pre vs POM4	<0.05*
EQ-5D-5L index values	Median	0.867	0.889	0.895	1.000	2.99	3, 42	<0.05*	Pre vs POM1	1.000
	IQR	0.797, 0.947	0.799, 0.895	0.882, 1.000	0.881, 1.000				Pre vs POM2	1.000
									Pre vs POM4	<0.05*
EQ-VAS	Median	70.0	80.0	80.0	90.0	3.93	3, 42	<0.05*	Pre vs POM1	1.000
	IQR	62.5, 80.0	60.0, 80.0	72.5, 85.0	77.5, 90.0				Pre vs POM2	0.889
									Pre vs POM4	<0.01*
IPAQ, METs·minutes/week	Median	528.0	792.0	990.0	990.0	1.42	3, 42	0.251	Pre vs POM1	1.000
	IQR	393.5, 1683.0	330.0, 981.0	729.0, 1666.0	546.5, 2970.0				Pre vs POM2	1.000
									Pre vs POM4	1.000
Sedentary time, minutes/day	Median	540.0	360.0	360.0	300.0	4.85	3, 42	<0.01*	Pre vs POM1	1.000
	IQR	360.0, 840.0	300.0, 600.0	270.0, 570.0	195.0, 360.0				Pre vs POM2	0.112
									Pre vs POM4	<0.01*

**Figure 3:**
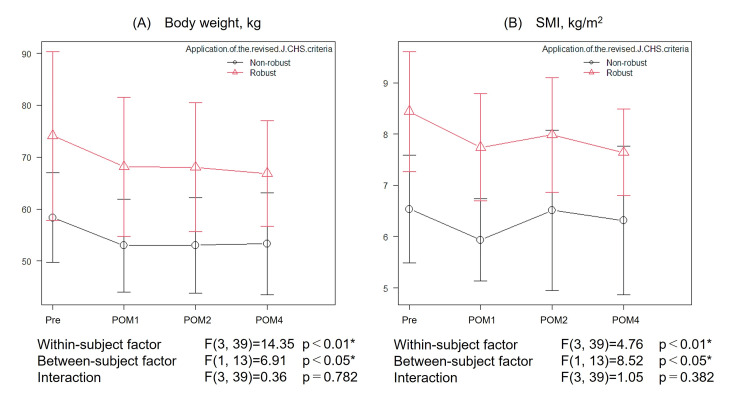
Changes in body composition over four months after kidney transplantation by preoperative frailty status (A) Body weight. (B) SMI. Line graphs showing the mean values and standard deviations of functional measures at four time points: in the preoperative period and at one, two, and four months after transplantation. Patients were stratified into the robust and non-robust groups. The p-values represent longitudinal changes (a within-subject factor), between-group differences (a between-subject factor), and interaction effects. Statistically significant findings (p < 0.05) are marked with asterisks. POM, postoperative month; Pre, preoperative period; SMI, skeletal muscle mass index; J-CHS: Japanese version of the Cardiovascular Health Study.

**Figure 4:**
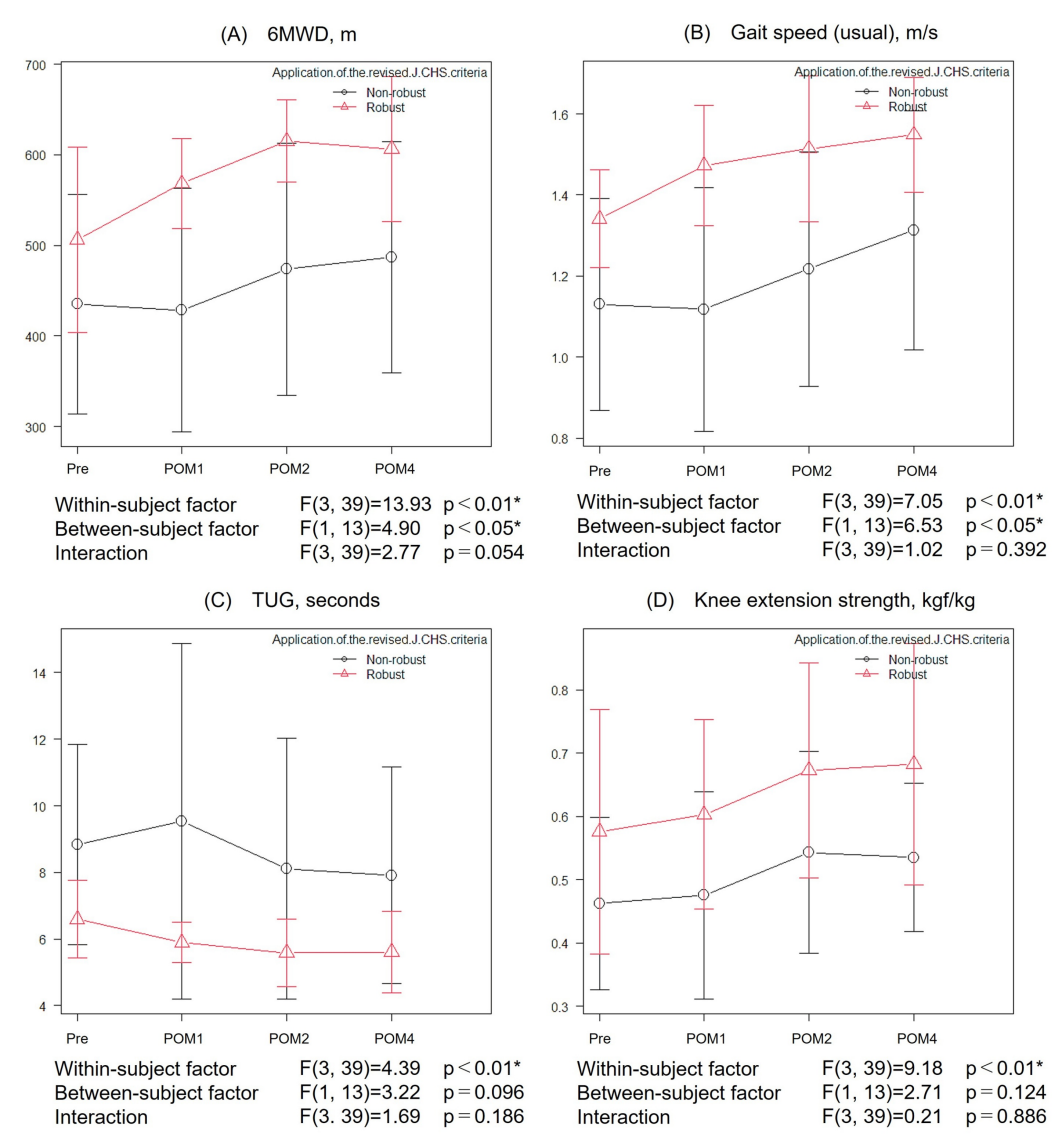
Changes in physical function over four months after kidney transplantation by preoperative frailty status (A) 6MWD, (B) gait speed (usual), (C) TUG, and (D) knee extension strength. Line graphs showing the mean values and standard deviations of functional measures at four time points: in the preoperative period and at one, two, and four months after transplantation. Patients were stratified into the robust and non-robust groups. The p-values represent longitudinal changes (a within-subject factor), between-group differences (a between-subject factor), and interaction effects. Statistically significant findings (p < 0.05) are marked with asterisks. 6MWD, 6-min walking distance; POM, postoperative month; Pre, preoperative period; TUG, Timed Up & Go test; J-CHS: Japanese version of the Cardiovascular Health Study.

Figures 3A, 3B, 4A-4D present the results of the two-way repeated measures ANOVA for body composition and physical function variables (body weight, SMI, 6MWD, gait speed, TUG, and knee extension strength) that showed significant longitudinal changes in the one-way repeated measures ANOVA. Significant between-group differences (a between-subject factor: robust group vs. non-robust group) were found in body weight, SMI, 6MWD, and gait speed. No significant between-group differences were observed for TUG and knee extension strength. There was a tendency toward an interaction effect, with the robust group showing a progressively greater improvement in 6MWD over time compared with the non-robust group; however, the result did not reach statistical significance (p = 0.054).

**Figure 5:**
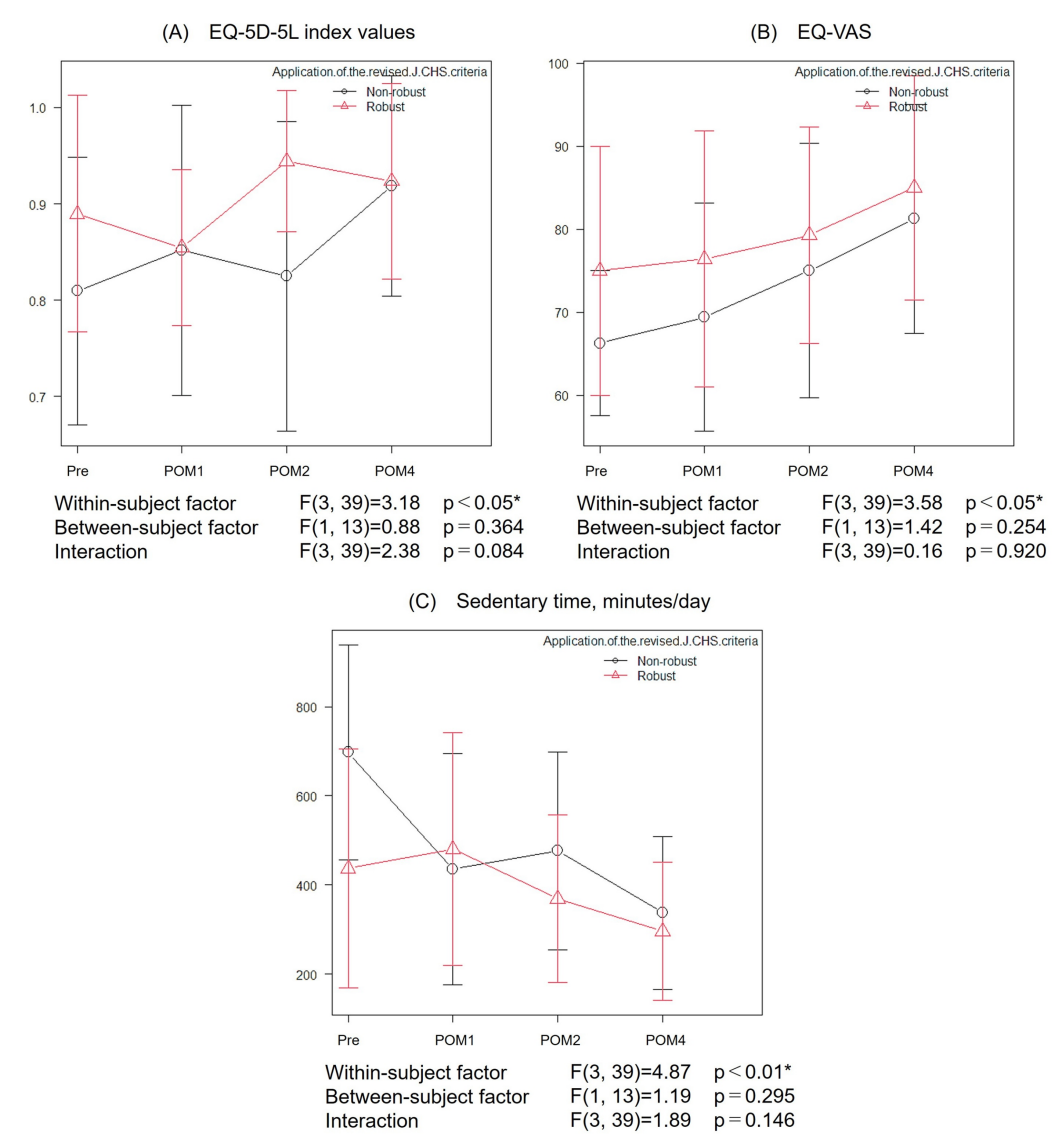
Changes in HRQOL and physical activity over four months after kidney transplantation by preoperative frailty status (A) EQ-5D-5L index values, (B) EQ-VAS, and (C) sedentary time. Line graphs showing the mean values and standard deviations of functional measures at four time points: in the preoperative period and at one, two, and four months after transplantation. Patients were stratified into the robust and non-robust groups. The p-values represent longitudinal changes (a within-subject factor), between-group differences (a between-subject factor), and interaction effects. Statistically significant findings (p < 0.05) are marked with asterisks. EQ-5D-5L, EuroQol 5-Dimension 5-Level; EQ-VAS, EQ visual analogue scale; HRQOL, health-related quality of life; POM, postoperative month; J-CHS: Japanese version of the Cardiovascular Health Study.

Figures 5A-5C present the two-way repeated measures ANOVA results for EQ-5D-5L index values, EQ-VAS, and sedentary time, all of which demonstrated significant longitudinal changes in the one-way ANOVA. Although within-subject improvements were observed across the postoperative period, no significant between-group differences (robust group vs. non-robust group) and no interaction effects were found.

## Discussion

This study investigated the time course of recovery of physical function and HRQOL following an early rehabilitation program after kidney transplantation, incorporating the influence of preoperative frailty. Despite a reduction in body weight and SMI after transplantation, improvements were observed in various physical functions and HRQOL, including 6MWD, gait speed, TUG, knee extension strength, EQ-5D-5L index values, EQ-VAS, and sedentary time (Table 2; Figure 3). Importantly, these improvements were also seen in patients classified as non-robust before transplantation, suggesting that early rehabilitation may be effective regardless of preoperative frailty status (Figures 4, 5).

A recent meta-analysis has demonstrated the effectiveness of aerobic and resistance exercise in patients more than a few months out from kidney transplantation, with improvements in physical function and HRQOL [6]. However, few studies have focused on the immediate postoperative recovery period. Therefore, we investigated functional recovery following early rehabilitation in these patients. Our findings suggest that the early rehabilitation program may have contributed to maintaining exercise tolerance during the vulnerable early postoperative phase. Exercise tolerance is known to be affected by reduced oxygen delivery due to conditions such as anemia and can decline before overt decreases in physical function are observed [20]. At one month after transplantation, although no significant improvements in physical function were detected, 6MWD was preserved despite the presence of postoperative anemia likely resulting from surgical stress and blood loss (Table 2). Furthermore, moderate-intensity exercise has been reported to have minimal adverse effects on renal perfusion [21]. By appropriately adjusting exercise intensity, the early rehabilitation program may have enabled safe implementation of exercise without compromising graft function during the immediate postoperative period. Importantly, all patients completed the program without interruption, confirming the efficacy and safety of early rehabilitation during this vulnerable period prior to discharge.

Kidney transplant recipients gradually resumed daily activities at around two months post-transplantation as graft function stabilized. Accordingly, the program transitioned to unsupervised home training after discharge, and our findings suggest that it may have contributed to attenuation of the progression of post-transplantation frailty. Improvements in 6MWD, knee extension strength, and TUG were observed at two months, consistent with the findings reported by Yamamoto et al. [8]. At four months, further improvements were seen in gait speed, EQ-5D-5L index values, EQ-VAS, and sedentary time. Furthermore, although body weight remained below preoperative levels, the reductions in SMI and increases in body fat percentage were relatively mild (Table 2). Kidney transplant recipients are prone to muscle atrophy and fat accumulation, particularly beyond three months post-transplantation [22]. While these improvements may have been influenced by stabilization of graft function and the accompanying resolution of frailty-related factors, such as malnutrition, uremia, chronic inflammation, mineral and bone disorders, cardiovascular complications, and insulin resistance [4], our findings suggest that continuing rehabilitation from the immediate postoperative period through the post-discharge phase may facilitate improvements in various aspects of physical function and HRQOL.

Our two-way repeated measures ANOVA showed that both the robust and non-robust groups demonstrated significant longitudinal improvements in 6MWD, gait speed, TUG, knee extension strength, EQ-5D-5L index values, EQ-VAS, and sedentary time during the two- to four-month post-transplantation period (Table 2; Figures 4, 5), indicating that early rehabilitation was effective regardless of preoperative frailty status. In clinical practice, robust patients often experience spontaneous recovery of physical function as graft function stabilizes. Indeed, 6MWD and gait speed, which were comparable between our two groups preoperatively, increased significantly in the robust group after transplantation. Although the interaction effect did not reach statistical significance, there was a trend toward greater longitudinal improvement in 6MWD in the robust group (p = 0.054). These findings are consistent with a previous report indicating that higher levels of physical activity after organ transplantation are associated with improved exercise tolerance and HRQOL [23]. Notably, significant functional gains were also observed in the non-robust group; these patients had significantly lower body weight, SMI, and grip strength and higher CCI scores preoperatively, indicating poorer overall health status (Table 1). Previous studies have found that recovery of peak oxygen uptake (VO₂) after kidney transplantation may take up to one year, and in some cases, functional impairments may persist in the medium to long term [24-26]. While these findings suggest a tendency toward delayed functional recovery in non-robust patients, the improvements observed in our non-robust group highlight the potential efficacy and broad applicability of early rehabilitation after kidney transplantation, even for patients with preoperative frailty. Given these functional improvements, it is important to consider their potential implications for long-term outcomes. The effects of exercise intervention on survival and graft prognosis in kidney transplant recipients remain unclear [6]. However, reduced physical function, including frailty, has been clearly associated with increased mortality [5]. The improvements in exercise tolerance, sedentary time, and EQ-VAS observed in this study reflect enhanced physical activity and HRQOL after kidney transplantation and may therefore have prognostic implications.

A significant correlation has been found between accelerometer-measured physical activity and the 6MWD in patients with CKD [27]. Although 6MWD improved in the present study, overall physical activity levels remained low after discharge (Table 2). This may be partly attributed to the characteristics of the IPAQ, which captures only activities performed in bouts of at least 10 minutes consecutively, making it less sensitive to short and intermittent physical activity. As a result, previous research has shown that physical activity levels are more significantly associated with sedentary time than with IPAQ scores [28,29]. Therefore, the reduction in sedentary time observed in the present study may reflect an increase in short-duration, low-intensity physical activity. Moreover, the natural recovery of physical activity after kidney transplantation has been reported to be gradual [26]. These findings underscore the importance of providing continued exercise guidance after discharge to help patients maintain and expand their engagement in physical activity. In response to modern rehabilitation needs, our program incorporates virtual reality-based training, which has been reported to be associated with high adherence and increased motivation for exercise in patients undergoing hemodialysis [30]. Nevertheless, owing to the retrospective nature of our study, we could not evaluate the individual effects of each component. Further research is needed to clarify their respective roles. However, beyond the use of mediVR KAGURA® device, incorporating virtual reality (VR) training and pedometer-based monitoring may help support sustained exercise participation, suggesting that this rehabilitation program could be applicable to other clinical settings.

This study has several limitations. First, it had a single-center retrospective design with a small sample size, which may limit the generalizability of its findings. However, given that a total of 1,782 kidney transplantations were performed in Japan in the 2022 fiscal year, the number of participants in this study cannot be considered extremely small in the national context. Although the sample size was limited to 15, consistent improvements were observed across most outcomes. Under typical assumptions (e.g., α = 0.05), large effect sizes (e.g., correlation coefficient ≥ 0.7) can often be detected even in small samples and may still provide meaningful clinical insights. Second, deceased-donor kidney transplant recipients were excluded because preoperative assessments could not be conducted. These patients typically experience prolonged wait times on hemodialysis or peritoneal dialysis and are more likely to be frail, so it is possible that the prevalence of frailty in our cohort was underestimated. Third, owing to perioperative fluctuations in health status, some patients had reduced training volume because of temporary interruptions, while others underwent prolonged hospitalization and received increased training volume. Therefore, the amount of training could not be fully standardized, and this variability should be considered when interpreting the findings. In addition, adherence to the home-based program could not be precisely quantified, as variability in pedometer records limited complete evaluation. Instead, adherence was indirectly assessed using IPAQ scores and sedentary time. Fourth, although there was a tendency toward greater improvement in 6MWD in the robust group, the p-value was borderline (p = 0.054), and this should be interpreted with caution as a trend rather than definitive evidence of group differences. Fifth, the follow-up period was limited to the first four months after transplantation, during which physical function is highly variable, and therefore, the longer-term trajectories of functional recovery and graft outcomes remain unclear. Finally, a control group was not included. This decision was based on ethical considerations, as it was deemed inappropriate to withhold rehabilitation from kidney transplant recipients who were willing to participate. Accordingly, our findings were interpreted in comparison with the existing literature.

## Conclusions

In this cohort, patients undergoing early rehabilitation after kidney transplantation demonstrated improved physical function and HRQOL over four months, with benefits observed across frailty groups. While causal inference is limited by study design, findings support the feasibility and potential value of continuous rehabilitation strategies. Furthermore, continued unsupervised training after discharge may contribute to sustained improvements in physical function and HRQOL during the post-discharge phase. These results may help inform the development of future rehabilitation programs based on systematic evaluations starting in the preoperative period.
